# Small interfering RNA pathway modulates persistent infection of a plant virus in its insect vector

**DOI:** 10.1038/srep20699

**Published:** 2016-02-11

**Authors:** Hanhong Lan, Haitao Wang, Qian Chen, Hongyan Chen, Dongsheng Jia, Qianzhuo Mao, Taiyun Wei

**Affiliations:** 1 Fujian Province Key Laboratory of Plant Virology, Institute of Plant Virology, Fujian Agriculture and Forestry University, Fuzhou, Fujian 350002, PR China

## Abstract

Plant reoviruses, rhabdoviruses, tospoviruses, and tenuiviruses are transmitted by insect vectors in a persistent-propagative manner. How such persistent infection of plant viruses in insect vectors is established and maintained remains poorly understood. In this study, we used rice gall dwarf virus (RGDV), a plant reovirus, and its main vector leafhopper *Recilia dorsalis* as a virus–insect system to determine how the small interference (siRNA) pathway modulates persistent infection of a plant virus in its insect vector. We showed that a conserved siRNA antiviral response was triggered by the persistent replication of RGDV in cultured leafhopper cells and in intact insects, by appearance of virus-specific siRNAs, primarily 21-nt long, and the increased expression of siRNA pathway core components Dicer-2 and Argonaute-2. Silencing of Dicer-2 using RNA interference strongly suppressed production of virus-specific siRNAs, promoted viral accumulation, and caused cytopathological changes *in vitro* and *in vivo*. When the viral accumulation level rose above a certain threshold of viral genome copy (1.32 × 10^14^ copies/μg insect RNA), the infection of the leafhopper by RGDV was lethal rather than persistent. Taken together, our results revealed a new finding that the siRNA pathway in insect vector can modulate persistent infection of plant viruses.

The outcome of host–pathogen interactions is highly variable and ranges from deleterious infection with lethal damage, persistent nonlethal infection with limited damage, to completely innocuous infections^1^. Insect–virus interactions are useful models with which to delineate persistent infection because many viruses that infect insects develop a persistent infection without obvious fitness costs to the insects^2^,^3^. In general, the persistent virus and insect use attack and counterattack machineries to reach an equilibrium at which viral infection is controlled but not eliminated in the insect vector^4^.

An insect small interfering RNA (siRNA) pathway has been shown to act as an antiviral immune pathway that is able to effectively modulate persistent replication of arthropod-borne animal viruses (arboviruses)^5^,^6^,^7^,^8^. A model for siRNA-mediated persistent infection of arboviruses in insects has been proposed^4^,^9^,^10^. During the replication process of arbovirus in the insect vectors, viral dsRNA genomes or dsRNA intermediates are recognized by a key component of the siRNA pathway, Dicer-2 (DCR2), to yield virus-derived siRNA (vsiRNA)^11^,^12^. These vsiRNAs reach the RNA-induced silencing complex (RISC) containing Argonaute-2 (AGO2) to specifically cleave viral mRNA^11^,^12^. The siRNA machinery may efficiently inhibit a lethal, acute infection to allow the virus to persist in the insect vector. In this way, both insect and virus progress into a metastable equilibrium that defines the state of persistent infection^4^,^9^,^10^.

Many plant viruses with an obvious agricultural impact such as reoviruses, rhabdoviruses, tospoviruses and tenuiviruses, can propagage persistently in their respective insect vectors^13^. Growing evidence has shown that the propagation of such plant viruses adversely affects the insect vectors. For example, the replication of southern rice black streaked dwarf virus (SRBSDV) and rice dwarf virus (RDV), two plant reoviruses, rice stripe virus (RSV), a tenuivirus, and impatiens necrotic spot virus, a tospovirus, can adversely affect life parameters such as survival, oviposition, hatchability and longevity of their respective insect vectors^14^,^15^,^16^,^17^,^18^. However, the propogative plant viruses only caused a limited adverse, rather than become pathogenic for their insect vectors^14^,^15^,^16^,^17^,^18^. How such persistent infection of plant viruses in their insect vectors is established and maintained remains poorly understood. Similar to the case of arboviruses, the siRNA response is also triggered by the replication of plant viruses in insect vectors^19^,^20^,^21^. Whether such a siRNA antiviral pathway is essential to the survival of insect vectors infected with plant viruses and, thus, the maintenance of these viruses in nature is unknown.

In this study, we used rice gall dwarf virus (RGDV), a plant reovirus, and its leafhopper vector *Recilia dorsalis* (Hemiptera: Cicadellidae) to determine how the siRNA antiviral pathway modulates the persistent infection by plant viruses of insect vectors. RGDV, first described in 1979 in Thailand, causes a severe disease of rice in southern China and Southeast Asia^22^,^23^,^24^,^25^. In recent years, RGDV has spread to Guangdong, Hainan and Guangxi Provinces of southern China^26^. RGDV is mainly transmitted in a persistent-propagative manner by the leafhopper vector with high efficiency, and the virus has evolved to adapt to the vector^26^,^27^,^28^. Both leafhopper *R. dorsalis* and its cultured cells support the multiplication of RGDV to a high titer and in both, a persistent and nonlethal infection develops^26^,^29^,^30^. We hypothesized that this persistence might have resulted from the control of viral replication below a pathogenic, deleterious threshold by the siRNA antiviral response, which is triggered by viral infection. Here, we demonstrated that knockdown of the DCR2 gene, the key siRNA pathway factor, effectively suppressed the accumulation of RGDV-derived siRNAs but obviously promoted the propagation of RGDV beyond the pathogenic threshold in the leafhopper *R. dorsalis*. Thus, for the first time, we revealed that the siRNA pathway modulates a metastable balance between viral accumulation and pathogenicity in the insect vector, allowing for viral persistence and highly efficient spread in nature.

## Results

### The siRNA pathway was triggered by the persistent infection of RGDV in leafhopper cells

**Figure 1:**
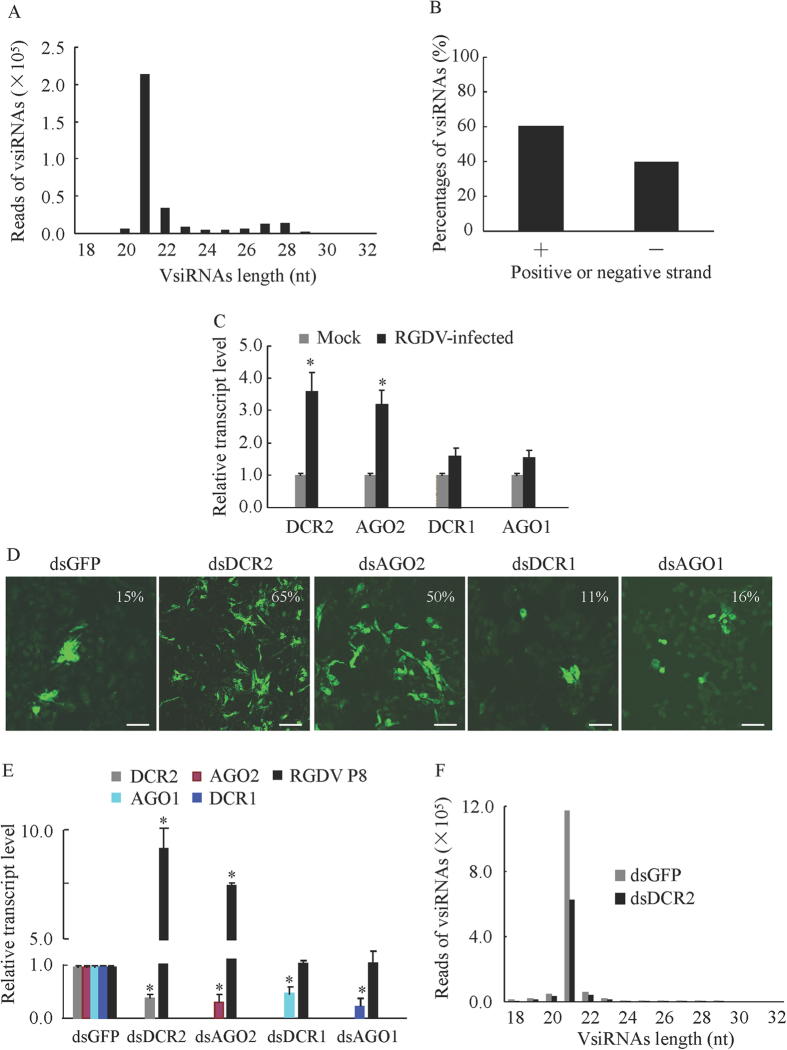
The siRNA pathway modulated the persistent infection by RGDV in VCMs. (**A**) Size distribution of vsiRNAs in VCMs inoculated with RGDV at 72 hpi. All reads in this analysis are redundant. (**B**) Percentage of vsiRNAs derived from the positive (+) and negative (−) strands of the RGDV genome in VCMs inoculated with RGDV at 72 hpi. All reads in this analysis were redundant. (**C**) Relative transcript abundance of DCR2, AGO2, DCR1 and AGO1 genes in mock-infected VCMs or RGDV-infected VCMs inoculated with RGDV at 72 hpi, as revealed by RT-qPCR assay, with mean ± standard deviation (SD) of three independent experiments. *P < 0.05. (**D**) Infection rates of VCMs transfected with the indicated dsRNAs at 72 hpi, as revealed by immunofluorescence microscopy. Bars, 25 μm. (**E**) Relative transcript abundance of the major outer capsid protein P8 gene of RGDV and RNAi-related genes in VCMs transfected with the indicated dsRNAs at 72 hpi by RT-qPCR assay with mean ± SD of three independent experiments. *P < 0.05. (**F**) The abundance of vsiRNAs in VCMs transfected with dsDCR2 or dsGFP at 72 hpi, as detected by small RNA deep sequencing. All reads in this analysis were redundant. Statistical analysis was conducted based on Tukey’s honest significant difference (HSD) test using SAS version IV (SAS Institute, Cary, NC, USA).

RNA interference (RNAi) has been considered as the most important antiviral response in *Drosophila* and mosquitoes^5^,^6^,^7^,^8^. Induction of the RNAi antiviral response is characterized by the production of siRNAs derived from viral genome^11^,^12^,^31^. Thus, we used small RNA deep sequencing to investigate the production of vsiRNAs in continuous cultures of the vector cells grown in monolayers (VCMs) derived from *R. dorsalis* after inoculation with RGDV at a multiplicity of infection (MOI) of 10^30^. In total, 1.18 × 10^7^ small RNA sequences were generated, of which 2.6% aligned to the RGDV genome (Table 1). The population of these vsiRNAs exhibited several main properties. The most dominant species was 21 nucleotides long (Fig. 1A). These vsiRNAs were divided approximately equally into positive (60%) and negative (40%) strands of the RGDV genome (Fig. 1B). The 21- and 22-nt vsiRNAs were distributed along the positive and negative genome strands with variable frequency into hot (high vsiRNA reads) and cold (low or no vsiRNA reads) spot distribution (Figure S1). These findings were consistent with vsiRNAs profiles in insect vectors infected with RSV and rice black streaked dwarf virus (RBSDV)^20^,^21^, strongly indicating that the dsRNA genome segments or its intermediates produced during the viral replication, rather than structured dsRNA regions of viral mRNAs, are the substrates for production of vsiRNAs by Dicer enzymes. Furthermore, as shown in Figure S2, a peak of vsiRNAs in the sizes ranging from 24 to 30 nt, resembling the group of PIWI-interacting RNAs (piRNAs) molecules, was identified (Figure S2A). These putative piRNAs were characterized by an enrichment for Uridine at position 1 (U1 bias) for antisense strands and an enrichment for Adenine at position 10 (A10 bias) for sense strands of these vsiRNAs (Figure S2B,C), which were implicated in recent small RNA profiling studies of mosquitoes^32^,^33^. Furthermore, the enrichment for U1 bias and A10 bias of piRNAs significantly different from the general nucleotide composition in 18–32 nt vsiRNAs (Figure S3), suggesting this two enrichments of piRNAs are specific. Identification of piRNAs requires a genetic dependence on PIWI-associated protein^32^,^33^, thus these small RNAs may be piRNAs. RNAi can mainly occur through the siRNA pathway or the micro-RNA (miRNA) pathway^6^. We identified two core components of the siRNA pathway, DCR2 and AGO2, and two core components of the miRNA pathway, Dicer-1 (DCR1) and Argonaute-1 (AGO1) from the leafhopper *R. dorsalis*. A real-time quantitative RT-PCR (RT-qPCR) assay showed that the transcript levels of the siRNA core components DCR2 and AGO2 increased about 3-fold after RGDV infection in VCMs at 72 h postinoculation (hpi) (Fig. 1C). However, the transcript levels of the miRNA components DCR1 and AGO1 increased about 1.5-fold (Fig. 1C). Thus, the RNAi pathway was triggered by the persistent infection of RGDV in insect vector cells.

To determine whether the RNAi pathway could modulate the persistent infection of RGDV in insect vector cells, the expression of DCR2, DCR1, AGO2 and AGO1 genes of leafhopper *R. dorsalis* were knocked down by transfection of the VCMs with synthesized dsRNAs targeting these genes or targeting GFP gene as a control *in vitro*. The VCMs were transfected with dsRNAs (dsDCR2, dsDCR1, dsAGO2, dsAGO1 or dsGFP) for 8 h, then inoculated with RGDV at a low MOI of 0.2 for 2 h. By 72 hpi, VCMs were treated with viral particle-specific IgG conjugated to fluorescein isothiocyanate (virus-FITC) to trace viral infection. We found that the treatment with dsDCR2 or dsAGO2 caused a significant increase in the percentage of infected cells from about 15% to 65% or from 15% to 50% when compared with dsGFP-treated cells (Fig. 1D). In contrast, treatment with dsDCR1 or dsAGO1 did not obviously alter the percentage of RGDV-infected cells (Fig. 1D). The RT-qPCR assay further indicated that the transfection with dsDCR2 or dsAGO2 increased viral RNA levels by about 8- or 6-fold, respectively (Fig. 1E). In contrast, no significant increase in viral RNA levels was observed for VCMs transfected with dsDCR1 or dsAGO1 (Fig. 1E). RT-qPCR assay also showed that the transfection of dsDCR2, dsDCR1, dsAGO2 or dsAGO1 caused an average of 70% reduction of the transcript levels of the respective targeted genes (Fig. 1E). Our results thus showed that the siRNA pathway, rather than the miRNA pathway could efficiently modulate the persistent infection of RGDV in its insect vector cells. Small RNA deep sequencing further confirmed that the knockdown of DCR2 led to a significant reduction of vsiRNAs production in virus-infected VCMs (Fig. 1F, Table 1). Thus, the knockdown of DCR2 expression suppressed the accumulation of vsiRNAs, but increased the propagation levels of RGDV in insect vector cells. Taken together, the core component DCR2 of the siRNA pathway is an efficient antiviral factor to control the replication of RGDV in its insect vector cells.

### The siRNA pathway controlled the persistent infection of RGDV in its leafhopper vector

**Figure 2:**
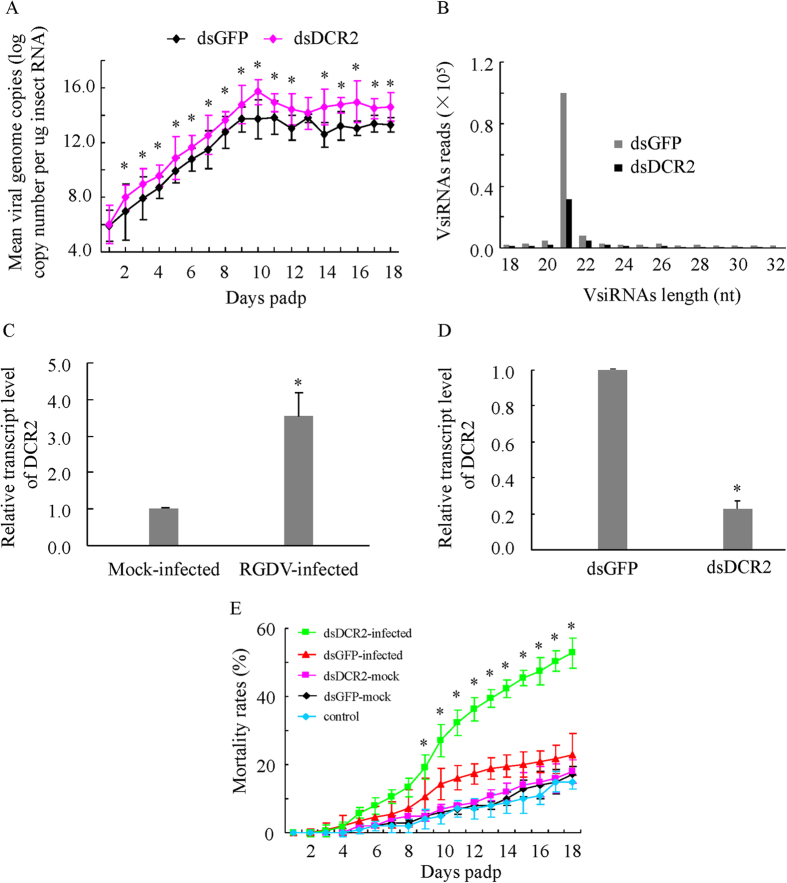
The siRNA pathway modulated the persistent infection of intact leafhoppers (*R. dorsalis*) by RGDV. (**A**) Mean viral genome copies of dsDCR2- or dsGFP-treated viruliferous leafhoppers from 1 day to 18 days padp, as determined by RT-qPCR assay with mean ±SD of three independent experiments. *P < 0.05. (**B**) Abundance of vsiRNAs from dsDCR2- or dsGFP-treated viruliferous *R. dorsalis* at 9 days padp, as detected by small RNA deep sequencing. All reads in this analysis were redundant. (**C**) Relative transcript abundance of DCR2 gene in RGDV-infected or mock-infected *R. dorsalis*, as determined by RT-qPCR assay, with mean ± SD of three independent experiments *P < 0.05. (**D**) Relative transcript abundance of the DCR2 gene in dsDCR2- or dsGFP-treated *R. dorsalis*, as determined by RT-qPCR assay, with means ± SD of three independent experiments *P < 0.05. (**E**) Mortality of dsGFP- or dsDCR2-treated *R. dorsalis* which had fed on uninfected or infected rice plants or of nontreated *R. dorsalis* which had fed on uninfected rice plants. Mortality was monitored daily for 18 days padp. Means ± SD from three biological replicates are shown. *P < 0.05. Statistical analysis was conducted based on Tukey’s honest significant difference (HSD) test using SAS version IV (SAS Institute, Cary, NC, USA).

To investigate whether the persistent RGDV infection in the leafhopper is modulated by the siRNA antiviral pathway, the dynamics of viral genome copies in the individual viruliferous leafhoppers (*n* = 380) receiving dsGFP or dsDCR2 were examined by RT-qPCR assay at different days post-first access to diseased plants (padp). The preliminary experiments generally showed more viral genome copies in dead viruliferous leafhoppers than in live viruliferous leafhoppers, and the mean viral genome copies remained relatively stable in live viruliferous leafhoppers during every stage of development regardless of the size of the leafhopper population (Figure S4). Thus, from 1 to 18 days padp, 10 live and all dead viruliferous leafhoppers were sampled daily to determine the viral genome copies for major outer capsid protein P8 gene of RGDV. RT-qPCR assay showed that the mean viral genome copies in dsGFP- or dsDCR2-treated viruliferous leafhoppers increased steadily from 1 to 10 days padp, and then remained stable from 10 to 18 days padp (Fig. 2A). The maximum mean viral genome copy was 6.69 × 10^13 ^copies/μg RNA from 17 dsGFP-treated viruliferous leafhoppers at 11 days padp (Fig. 2A). However, the maximum mean viral genome copy had increased to 5.21 × 10^15 ^copies/μg RNA from 40 dsDCR2-treated viruliferous leafhoppers at 10 days padp (Fig. 2A). Thus, the maximum mean viral genome copy of dsDCR-treated group was about 100-fold higher than in the dsGFP-treated group. When the same numbers of leafhoppers harvested at same timepoints were tested, the mean viral genome copies were also significantly higher in dsDCR2-treated viruliferous leafhoppers than dsGFP-treated viruliferous leafhoppers from 9 to 13 days padp (Figure S5). We then used immunofluorescence microscopy to compare the distribution patterns of RGDV accumulation between dsGFP- and dsDCR2-treated leafhoppers. A more intense and extensive fluorescence of RGDV antigens was observed in the midguts or salivary glands from dsDCR2-treated leafhoppers (Figure S6), confirming that dsDCR2 treatment significantly increased viral accumulation in the leafhoppers.

We then investigated the ability of dsDCR2 treatment to suppress the accumulation of vsiRNAs during virus replication in the leafhopper. Small RNA deep sequencing showed that the length distribution patterns of the vsiRNAs derived from dsGFP- or dsDCR2-treated leafhoppers were similar to that derived from the cultured cells at 9 days padp (Fig. 2B). However, the production of vsiRNAs derived from dsDCR2-treated leafhoppers was reduced by approximately 64% compared with that from dsGFP-treated controls at 9 days padp (Fig. 2B, Table 1). Furthermore, RT-qPCR assay further indicated that the transcript level of the DCR2 gene was about 3.5-fold higher in the viruliferous leafhopper than in the nonviruliferous control (Fig. 2C), but the transcript level of the DCR2 gene was reduced by about 70% in the dsDCR2-treated group compared with the dsGFP-treated control (Fig. 2D). Our results showed that the knockdown of the expression of DCR2 effectively suppressed the production of vsiRNAs, increased the propagation ability of RGDV in its leafhopper vector.

Leafhopper mortality assay was performed to determine the effects of increased viral accumulation on insect mortality. Our test showed that about 18% of dsGFP- or dsDCR2-treated nonviruliferous leafhoppers were dead at 18 days padp (Fig. 2E). This represented the normal mortality of leafhoppers during the development stages. Thus, the silencing of DCR2 did not affect the normal mortality of nonviruliferous leafhopper. We further tested whether the silencing of DCR2 affected the mortality of the viruliferous leafhopper. Significantly higher mortality was observed from 9 to 18 padp for dsDCR2-treated viruliferous leafhoppers than for dsGFP-treated viruliferous leafhoppers (Fig. 2E). At 18 days padp, about 53% of dsDCR2-treated leafhoppers were dead, compared with about 23% of dsGFP-treated leafhoppers (Fig. 2E). Thus, RGDV infection caused a limited mortality of dsGFP-treated viruliferous leafhoppers, but caused a high mortality of dsDCR2-treated viruliferous leafhoppers.

**Figure 3:**
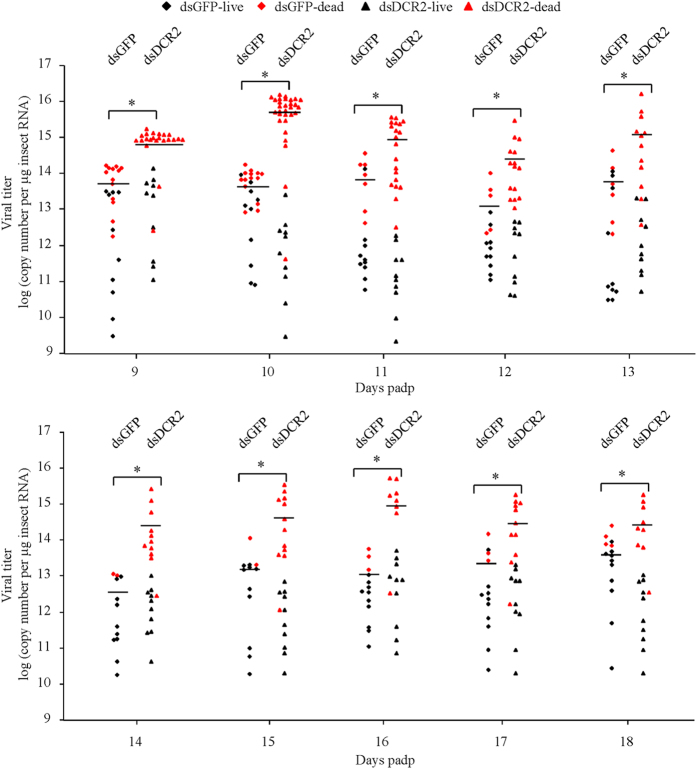
Mortality of leafhoppers was associated with increased viral loads induced by silencing of DCR2. Second-instar nymphs of *R. dorsali* (*n* = 380) were microinjected with dsDCR2 or dsGFP, then allowed to feed on RGDV-infected rice plants for 1 day. Viral genome copies were determined by RT-qPCR for individual insects and calculated as the log of the copy number/μg insect RNA from 10 live insects and total dead insects daily from 9 to 18 days padp. Each data point represents an individual insect. Horizontal lines represent the mean viral genome copies for each data set. Black dots indicate live insects, red dots dead insects. *P < 0.05. Statistical analysis was conducted based on Tukey’s honest significant difference (HSD) test using SAS version IV (SAS Institute, Cary, NC, USA).

We then determined the threshold of viral accumulation required for insect mortality. We analyzed the viral genome copies from individual insects daily from 9 to 18 days padp. We totally analyzed 250 (100 live and 150 dead) dsDCR2-treated leafhoppers and 159 (100 live and 59 dead) dsGFP-treated leafhoppers. We found that the viral genome copies for 200 live leafhoppers ranged from 2.90 × 10^9^ to 1.32 × 10^14 ^copies/μg insect RNA, and for 209 dead leafhoppers ranged from 4.07 × 10^11^ to 1.63 × 10^16 ^copies/μg insect RNA (Fig. 3, Table S1). The 13 dsGFP-treated and 106 dsDCR2-treated leafhoppers with viral genome copies higher than the maximum viral genome copy of live leafhoppers (1.32 × 10^14 ^copies/μg insect RNA) were dead, accounting for 22.0% of the total dsGFP-treated dead leafhoppers and 70.7% of the total dsDCR2-treated dead leafhoppers, respectively (Fig. 3, Table S1). Because 1.32 × 10^14 ^copies/μg insect RNA was the maximum level of viral accumulation found in live leafhoppers, we deduced that this viral genome copy was very close to the mortality threshold. Below this putative mortality threshold, 19.1% of the total dsGFP-treated leafhoppers and 17.6% of the total dsDCR2-treated leafhoppers were dead (Fig. 3, Table S2). Under normal development condition, about 18% of adult nonviruliferous leafhoppers were dead (Fig. 2E). These results suggested that the mortality of dsGFP- or dsDCR2-treated leafhoppers with viral genome copies below the putative mortality threshold belonged to the natural death of insect developments. Thus, when the viral accumulation level rose above a certain threshold of viral genome copy (1.32 × 10^14^ copies/μg insect RNA), the infection of the leafhopper by RGDV was lethal rather than persistent. Our result clearly showed that the silencing of DCR2 dramatically increased viral accumulation, which finally caused high mortality of leafhoppers. Thus, the virus-associated mortality of leafhoppers was dose-dependent. Moderate viral accumulation led to limited mortality among the leafhopper vectors, but a large increase in viral accumulation after the silencing of DCR2 easily killed all leafhopper vectors.

### Cytological changes in virus-associated leafhoppers caused by dsDCR2 treatment

**Figure 4:**
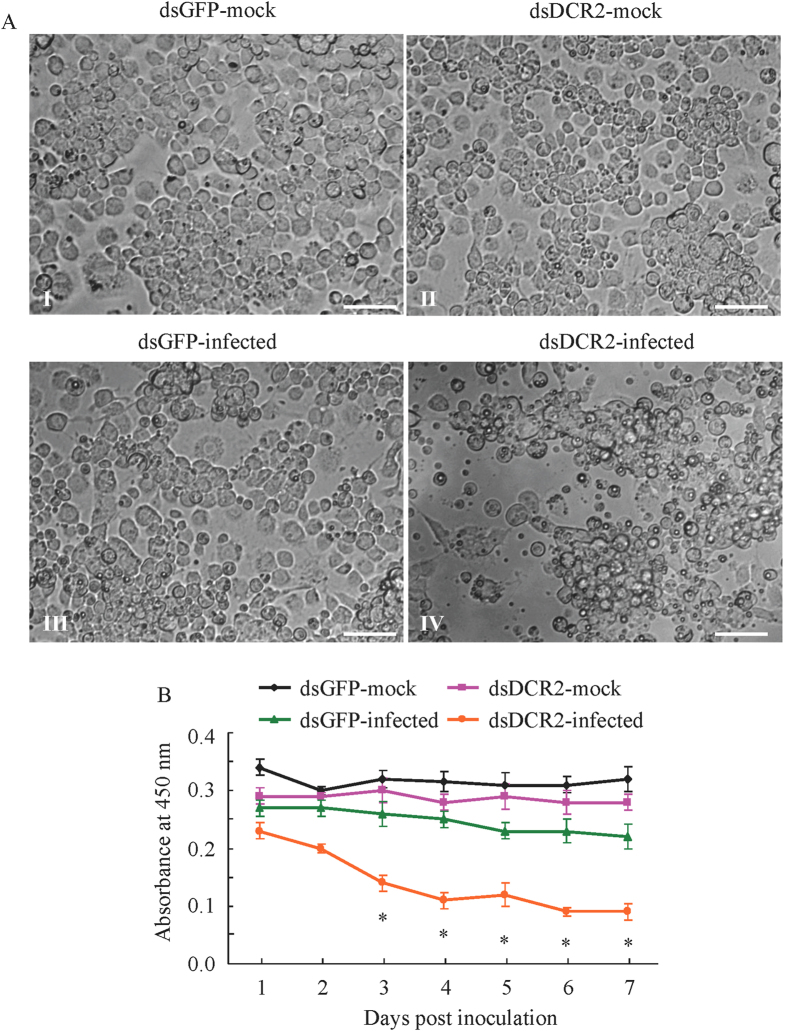
Cytopathological changes caused by the increase in viral accumulation in dsDCR2-treated VCMs derived from leafhoppers. (**A**) Bright-field images of cell morphology of mock-infected VCMs treated with dsGFP (panel I) or dsDCR2 (panel II), and virus-infected VCMs treated with dsGFP (panel III) or dsDCR2 (panel IV). Bars, 500 μm. (**B**) Absorbance at 450 nm of RGDV-infected VCMs treated with dsDCR2 or dsGFP, or mock-infected VCMs treated with dsDCR2 or dsGFP as determined in a cell viability assay, with means ± SD of three independent experiments. *P < 0.05. Statistical analysis was conducted based on Tukey’s honest significant difference (HSD) test using SAS version IV (SAS Institute, Cary, NC, USA).

We examined any cytopathological changes caused by the increase in viral accumulation in dsDCR2-treated leafhoppers. We first used cultured cells derived from *R. dorsalis* to investigate the cytopathological changes caused by the increase. Treatment with dsDCR2 resulted in severe cytopathological changes in RGDV-infected cultured cells (Fig. 4A), such as cell clumping, loss of confluent monolayers, and formation of cytoplasmic vacuoles (Fig. 4A). In contrast, RGDV-infected cells treated with dsGFP were morphologically similar to dsGFP- or dsDCR2-treated uninfected cells, showing the typical size and confluent morphology of monolayers (Fig. 4A). In addition, a cell viability assay for testing cell proliferation showed that absorbance at 450 nm of RGDV-infected cells treated with dsGFP decreased only by 23% when compared with the dsGFP- or dsDCR2-treated uninfected cells over 7 days (Fig. 4B). However, dsDCR2 treatment caused a significant decrease by 70% in the absorbance in RGDV-infected cells (Fig. 4B), suggesting that the viability of *R. dorsalis* cells was greatly affected by increased viral accumulation induced by the silencing of DCR2.

**Figure 5:**
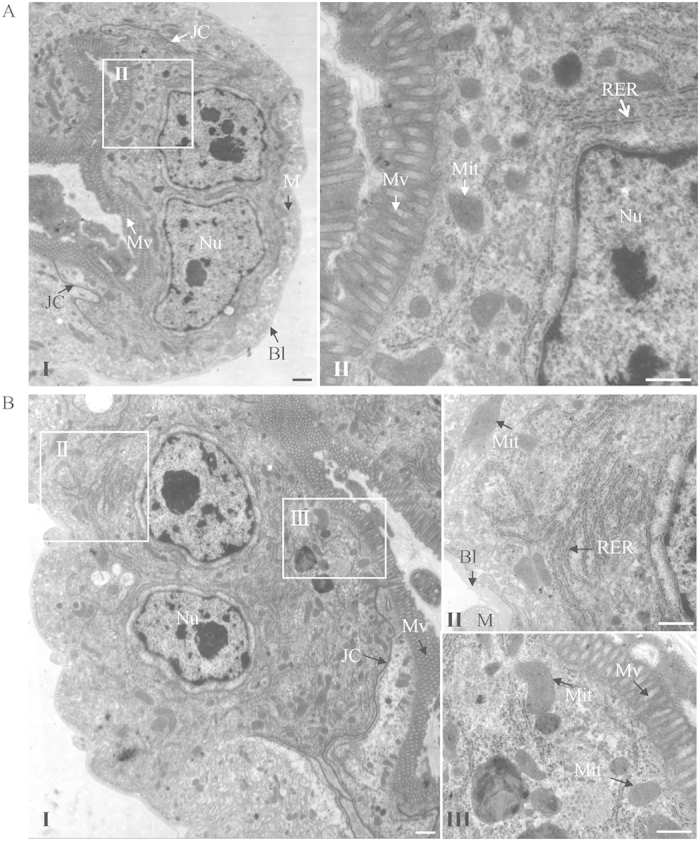
Treatment of dsDCR2 had no adverse effect on the cytology of midgut epithelial cells of the nonviruliferous leafhopper vectors. Transmission electron micrographs of midgut epithelial cells from leafhoppers treated with dsGFP (**A**) or dsDCR2 (**B**) showing normal histology and ultrastructure including nucleus, microvilli, mitochondrion, rough endoplasmic reticulum, junctional complexes, muscle, and basal lamina. Panels II and III are enlarged images of the boxed areas in panel I. BL, basal lamina; M, muscle; Mv, microvilli; Mit, mitochondrion; Nu, nucleus; JC, junctional complexes; RER, rough endoplasmic reticulum. Bars, 2 μm (I), 500 nm (II and III).

**Figure 6:**
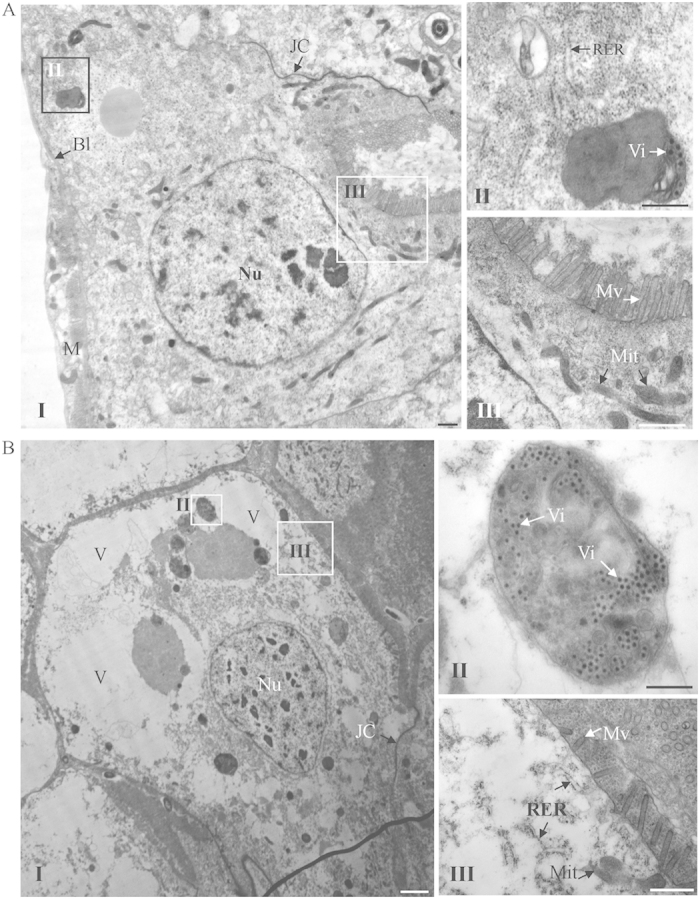
Cytopathological changes in midgut epithelial cells of dsGFP- or dsDCR2-treated viruliferous leafhoppers. The cytopathological effects included vacuolization of cytoplasm, reduction of cytoplasmic density, loss of microvilli brush border integrity, and degeneration of mitochondria and rough endoplasmic reticulum. The degree of cytopathogical changes were slight in dsGFP-treated leafhopper (**A**) but were severe in dsDCR2-treated leafhopper (**B**). Panels II and III are enlarged images of the boxed areas in panel I to show the degree of cytopathological effects. BL, basal lamina; JC, junctional complexes; M, muscle; Mit, mitochondrion; Mv, microvilli; Nu, nucleus; RER, rough endoplasmic reticulum; V, vacuole; Vi, virions. Bars, 2 μm (I), 500 nm (II and III).

We then used electron microscopy to observe cytopathological changes in the midgut epithelial cells caused by the increase of viral accumulation after dsDCR2 treatment. Cells in the dsDCR2-treated and dsGFP-treated nonviruliferous leafhopper had normal histology and ultrastructure, suggesting that silencing of the DCR2 gene had no adverse effect on the cytology of the midgut epithelial cells of the nonviruliferous leafhoppers (Fig. 5). In contrast, in dsDCR2-treated viruliferous leafhopper, severe cytological and ultrastructural abnormalities were observed in the midgut epithelial cells, including vacuolization in the cytoplasm, reduction of cytoplasmic density, the loss of microvilli brush border integrity, and the degeneration of mitochondria and rough endoplasmic reticulum (Fig. 6). Although the dsGFP-treated viruliferous leafhopper also contained these abnormalities, the severity was lower than in dsDCR2-treated viruliferous leafhoppers (Fig. 6). Midgut epithelial cells of dsDCR2-treated viruliferous leafhoppers seemed to be undergoing necrosis and degenerating. Our observation of cytopathological changes for virus-infected cultured cells and insect midgut epithelial cells caused by dsDCR2 treatment agreed with the observation that the silencing of DCR2 easily killed leafhopper vectors^34^. The high pathogenicity of RGDV in dsDCR2-treated leafhoppers finally affected viral transmissibility ability. At 15 days padp, a transmission test showed that about 75% of dsGFP-treated leafhopper could actually transmit the virus (Figure S7). In contrast, only about 50% of dsDCR2-treated leafhoppers had transmissibility ability (Figure S6).

## Discussion

Almost 80% of plant viruses that cause serious agricultural losses are transmitted by vector insects^35^,^36^. Plant viruses such as tospoviruses, tenuiviruses, rhabdoviruses and reoviruses, are transmitted by their respective insect vectors in a persistent-propagative manner^13^. These viruses are maintained in nature through alternating virus replication in susceptible plant hosts and insect vectors. While infection of the plant host is typically associated with pathology and disease, a persistent nonlethal infection is established in the insect vector^2^,^3^. Currently, the mechanisms permitting this persistence are poorly understood. Here, we used a plant reovirus RGDV and its main leafhopper vector as a model to better understand how the siRNA pathway modulates the persistent infection of a plant virus in its insect vector. The replication of RGDV in the continuously cultured cells derived from leafhoppers and in the intact insect was targeted by the siRNA machinery as shown by the presence of vsiRNAs primarily 21-nt long (Figs 1 and 2). The transcript levels of genes for core components DCR2 and AGO2 of the siRNA pathway were significantly upregulated after viral infection (Figs 1 and 2). Furthermore, the knockdown of the expression of DCR2 strongly suppressed the production of vsiRNAs and promoted the propagation ability of RGDV (Figs 1, 2, 3). Thus, the persistent infection of RGDV in its insect vector triggered a strong siRNA antiviral response resulting in the accumulation of vsiRNAs. Our results revealed a new finding that this siRNA antiviral response can efficiently control RGDV replication below a putative cytopathogenic threshold, thus playing an important role in the establishment and maintenance of the persistent infection of RGDV in its insect vector. Because persistent-propagative plant viruses are not generally associated with detrimental effects in their natural insect vectors and the replication of such plant viruses may trigger an antiviral siRNA response in insect vectors, our results have potentially broad implications for understanding how the persistent infection of plant viruses in insect vectors in nature are established.

Considering these points, we propose a model to account for the persistent infection of plant viruses in insect vectors modulated by the insect siRNA antiviral pathway. However, there is one key unanswered question. Have plant viruses evolved to encode viral suppressors of RNA silencing (VSRs) in the insect vectors? In an evolutionary arms race between insect and viral pathogen, some arboviruses have evolved to encode VSRs in insect vectors^11^,^12^. The expression of VSRs by arboviruses in the vector seems to be compatible with the establishment of persistence. For example, expression of the Flock house virus (FHV) B2 protein, a VSR, from recombinant alphaviruses dramatically alters the pathogenic outcome of the virus infection in a mosquito host, resulting in rapid and complete mortality^9^,^10^. Whether or not VSRs encoded by plant viruses in host plants have similar functions in insect vectors has been the subject of debate. The expression of the VSRs, NSs protein of tomato spotted wilt virus, a tospovirus, and NS3 protein of rice hoja blanca virus, a tenuivirus, facilitates the replication of baculovirus in nonhost Sf9 cells^37^,^38^, and these results suggest that the VSRs encoded by plant viruses might play a similar role during virus infection in the insect vectors. The nonstructural proteins Pns11 and Pns12 of RGDV are the VSRs in plant hosts^39^,^40^. Furthermore, Pns11 is responsible for the formation of virus-containing tubules to facilitate viral spread in insect vector, and Pns12 is one of the components of the viroplasm matrix, the site for viral replication and assembly of progeny virions^30^,^41^. The highly efficient persistent-propagation of RGDV in its leafhopper vector likely requires a balance against VSR potency and the antiviral response of the insect vector. From this point, RGDV might encode a less potent VSR for minimizing the negative impact on vector fitness to allow the control of excessive viral accumulation by the siRNA antiviral pathway. Further experiments are needed to determine whether RGDV Pns11 or Pns12 acts as such a VRS in its insect vector.

However, we have a clear answer for the question why the silencing of DCR2 can easily kill viruliferous leafhopper vectors. RGDV infection causes limited pathology and mortality of leafhopper vectors (Figs 2 and 3), suggesting that viral infection is unfavorable for the expansion of the *R. dorsalis* population. Because persistent-propagative plant viruses exclusively depend on the vectors for viral replication and spread, it is easy to understand that they can cause damage similar to insect pathogens. Here, we show that RGDV becomes highly pathogenic to leafhopper when the core component DCR2 of the siRNA pathway is silenced during viral infection (Figs 2, 3 and 6). Because the silencing of DCR2 did not affect insect development or cause any cytopathological changes in the absence of viral infection (Figs 2 and 5), we determined that the increase in viral accumulation after the silencing of DCR2 directly caused the serious cytopathological changes (Figs 2, 3 and 6), which finally led to high mortality of the leafhoppers. Thus, our results clearly showed that the pathogenicity of RGDV is correlated with its accumulation levels in leafhopper vectors. Our results demonstrated that, as long as viral accumulation exceeded the viral genome copies of 1.32 × 10^14 ^copies/μg insect RNA, RGDV infection would kill leafhopper vectors (Fig. 3 and Table S1). We thus determined that this level (1.32 × 10^14 ^copies/μg insect RNA) was very close to the mortality threshold of viral accumulation level in leafhopper vectors. Thus, if viral accumulation level is below this threshold, persistent viral infection is established, and if the level of viral accumulation exceeds this threshold, the infection becomes lethal. RGDV can be transmitted by leafhopper vectors with high efficiency^26^. There appears to be metastable balance efforts from both virus and insect to regulate viral accumulation close to the putative mortality threshold, so that the virus can persist in insects without causing significant adverse effects, allowing the virus to be transmitted with high efficiency.

Persistent-propagative viruses must be transmitted between insect vectors and plant hosts to be maintained in nature and thus must optimize their transmission potential to ensure their continuity. For the first time, we revealed that, from an evolutionary standpoint, an insect vector has developed a mechanism, by triggering a siRNA antiviral response, to optimize the delicate balance between robust replication and limited pathology of a virus in its insect vector, allowing for persistent infection and highly efficient vectoring in nature.

## Methods

### Insects, cells, virus and antibody

Leafhoppers (*R. dorsalis*) were collected from Guangdong Province in southern China, reared on young rice plants and kept in cages in a plant growth chamber at 28 °C and 80% relative humidity with 15 h light/9 h dark. VCMs derived from *R. dorsalis* were maintained in Kimura’s insect medium at 25 °C as described previously^30^. RGDV was collected from Guangdong Province and purified from infected rice plants as described previously^42^,^43^. Rabbit polyclonal antibody against intact viral particles of RGDV was conjugated directly to FITC or rhodamine (Invitrogen, USA) according to the manufacturer’s instructions, as described previously^43^,^44^.

### Identification of RNAi-related genes and their transcript abundance during viral infection in VCMs

To obtain the sequences encoding the core components of the RNAi pathway in the transcriptome of *R. dorsalis*, we used a local BLASTN search with sequences of *N. lugens* (JX023532.1, JX644040.1, KC316038.1 and JX644040.1 for DCR2, DCR1, AGO2 and AGO1, respectively) as queries^45^. The obtained gene sequences for DCR2, DCR1, AGO2 and AGO1 of *R. dorsalis* were deposited in GenBank with accession numbers KT191018, KT191019, KT191020 and KT191021, respectively.

VCMs were inoculated with RGDV at a MOI of 10 for 2 h as described previously^43^,^44^. Total RNA was extracted with Trizol reagent (Invitrogen, USA) according to the manufacturer’s instructions at 72 hpi. The relative transcript abundance of RNAi pathway genes in RGDV-infected or mock-infected VCMs was estimated using RT-qPCR with the SYBR Green PCR MasterMix kit (Promega, USA) with the primers in Table S2, as described previously^46^. The relative abundance of the RNAi pathway genes was normalized to an internal control gene actin and estimated by the 2^−ΔΔCt^ (cycle threshold) method.

### Knockdown of RNAi-related genes expression in VCMs

PCR products of the segments of RNAi-related genes including 562 bp for DCR2, 482 bp for DCR1, 552 bp for AGO2 and 583 bp for AGO1 were used as template for dsRNA synthesis (dsDCR2, dsDCR1, dsAGO2 and dsAGO1) *in vitro* using a T7 RiboMAX Express RNAi System kit (Promega, USA) with the primers in Table S2 as described previously^42^,^44^. The 630 bp segment of the GFP gene was used for *in vitro* dsRNA synthesis (dsGFP) as the control.

VCMs were treated with dsRNAs (0.1 μg**/**μl) in the presence of the Cellfectin reagent (Invitrogen, USA) for 8 h and then inoculated with RGDV at a low MOI of 0.2 for 2 h as described previously^42^,^44^. At 72 hpi, immunofluorescence confocal microscopy was used to detect viral infection by virus-FITC, as described previously^42^,^43^,^44^. In addition, total RNA was extracted from VCMs with Trizol reagent (Invitrogen, USA) for RT-qPCR assays using the primers in Table S2 and the SYBR Green PCR MasterMix kit (Promega, USA). The relative abundance of RNAi-related genes and the major outer capsid protein P8 gene of RGDV were normalized to an internal control gene actin and estimated by the 2^−ΔΔCt^ (cycle threshold) method as described above.

### Knockdown of DCR2 gene expression in leafhopper vector

Total 380 second-instar nymphs of *R. dorsali* were microinjected with dsDCR2 or dsGFP (0.1 μg**/**μl) using Nanoject II Auto-Nanoliter Injector (Spring, USA) as described previously^47^, then fed on diseased plants for 1 day. At different days padp, ten live and total dead leafhoppers were individually sampled daily for 18 days. Total RNA was extracted from individual leafhoppers with Trizol reagent (Invitrogen, USA). The cDNAs were synthesized using gene-specific primers of the major outer capsid protein P8 gene of RGDV in Table S2. RT-qPCR was performed using the SYBR Green PCR MasterMix kit (Promega, USA) as described above. Cycle thresholds (CT) were obtained by the RT-qPCR assay. The viral genome copy was calculated as the log of the copy number per microgram of insect RNA by mapping the CT value to the standard curve of the major outer capsid protein P8 gene of RGDV (*y* = −3.06*x* + 54.44). In addition, the relative abundance of DCR2 gene was confirmed by RT-qPCR as described already. Furthermore, midguts and salivary glands of viruliferous leafhoppers treated with dsGFP or dsDCR2 (n = 100) were dissected, immunolabelled with viral particle-specific IgG conjugated to rhodamine (virus-rhodamine) and the actin dye phalloidin-FITC (Invitrogen, UAS), and detected by immunofluorescence microscopy at 5 or 10 days pads as described previously^26^.

### Viral transmission by leafhopper that had received dsRNAs

One hundred second-instar nymphs of leafhoppers were microinjected with dsDCR2 or dsGFP. The microinjected leafhoppers were fed on RGDV-infected rice plants for 1 day, and then placed on healthy rice seedlings. At 12 days padp, an individual insect was fed on a healthy rice seedling for 2 days. The seedlings were grown longer for observing the disease symptoms. Total RNA was also extracted from inoculated seedlings to determine the presence of transcript for the RGDV P8 gene with primers in Table S2 to calculate the transmission rates.

### Small RNA sequencing

Total RNAs from VCMs or leafhopper vectors were extracted using Trizol reagent (Invitrogen, USA). Small RNAs were sequencing by Solexa/Illumina system at the Wuhan Genomics Institute at Shenzhen (BGI Shenzhen, China) as described previously^20^,^21^. Sequences of 18–32 nt small RNAs were mapped to the RGDV genome (http://www.ncbi.nlm.nih.gov/genome/?term=rice+gall+dwarf+virus). Only small RNA reads perfectly matched to the RGDV genome were analyzed. All raw small RNA sequences have been deposited in NCBI Sequence Read Archive under the accession number of SRP066208.

### Cell morphology and viability assay

VCMs were plated into each well of a 96-well plate and cultured for 24 h, then transfected with dsGFP or dsDCR2 (0.1 μg/μL) in the presence of the Cellfectin reagent (Invitrogen, USA) for 8 h, inoculated with RGDV at a low MOI of 0.2 for 2 h and photographed 7 days post inoculation using an Olympus BX51 microscope (Olympus, Japan). In addition, the cell viability assay using Cell Counting Kit-8 (CCK-8; Sigma-Aldrich, USA) were used to evaluate the effects of elevated viral replication induced by DCR2 silencing on VCM viability. At different days after inoculation, 10 μL of CCK-8 solution was added to each well and incubated for 3 h, and a microplate reader was used to measure absorbance at 450 nm.

### Transmission electron microscopy

Second-instar nymphs of *R. dorsalis* were microinjected with dsDCR2 or dsGFP (0.1 μg/μL) using a Nanoject II Auto-Nanoliter Injector (Spring, USA), then fed on diseased rice plants for 1 day. The midguts were excised, fixed, dehydrated, and embedded, and thin sections were cut and examined with a H-7650 Hitachi transmission electron microscope at 80 KV as described previously^43^,^44^.

### Statistical analysis

Percentage data were arcsine square-root-transformed before the analysis. Multiple comparisons of the means were conducted based on Tukey’s honest significant difference (HSD) test using SAS version IV (SAS Institute, Cary, NC, USA). Differences between samples were regarded as significant at *P* < 0.05.

## Additional Information

**How to cite this article**: Lan, H. *et al*. Small interfering RNA pathway modulates persistent infection of a plant virus in its insect vector. *Sci. Rep*. **6**, 20699; doi: 10.1038/srep20699 (2016).

## Supplementary Material

Supplementary Information

## Figures and Tables

**Table 1:** Silencing of DCR2 gene suppressed accumulation of siRNAs derived from the RGDV genome.

Category	VCMs			Leafhopper vector	
	Control	dsGFP	dsDCR2	dsGFP	dsDCR2
Total small RNA reads^a^	11835372	11057285	11509794	13769943	16327254
Unique small RNA reads^a^	2446143	1576542	1856333	4607665	5049873
Total RGDV matched reads^b^ (%^c^)	307968 (2.6%)	1375897 (12%)	935630 (8%)	134678 (1%)	48623 (0.2%)
Unique RGDV matched reads^b^ (%^c^)	42447 (1.7%)	56906 (3.6%)	48242 (2.1%)	30225 (0.7%)	16188 (0.3%)

^a^Number of small RNA sequences (18–32 nt).

^b^Number of viral siRNAs perfectly matched to the RGDV genome.

^c^Percentage of viral siRNAs perfectly matched to the RGDV genome.
