# Supplementary material for: Small interfering RNA pathway modulates persistent infection of a plant virus in its insect vector
Source: Sci Rep. 2016 Feb 11;6:20699. doi: 10.1038/srep20699 (PMC4750021; doi:10.1038/srep20699)
Supplement: Supplementary Information [file srep20699-s1.pdf]

Small interfering RNA pathway modulates persistent infection of a plant virus in its insect vector

Hanhong Lan[#], Haitao Wang[#], Qian Chen[#], Hongyan Chen, Dongsheng Jia, Qianzhuo Mao, and Taiyun Wei[*]

Fujian Province Key Laboratory of Plant Virology, Institute of Plant Virology, Fujian Agriculture and Forestry University, Fuzhou, Fujian 350002, PR China

*Author to whom all correspondence should be addressed, as follows:

Dr. Taiyun Wei, Institute of Plant Virology, Fujian Agriculture and Forestry University, Fuzhou, Fujian 350002, PR China

Tel: +86-591-83789270. Fax: +86-591-83789439.

E-mail: weitaiyun@fafu.edu.cn

Supplementary Figure Legends

Figure S1 Frequency distribution of 21- and 22-nt vsiRNAs to positive and negative strands of RGDV genome in VCMs at 72 hpi. The *y* axis shows the reads of the 21- and 22-nt vsiRNAs mapping to the corresponding nucleotide position on the *x* axis. Positive and negative numbers represent the reads of vsiRNAs mapping to the positive and negative strand, respectively. RGDV S1-S12 represents the twelve RNA segments of RGDV genome. Black and green lines denote 21-nt vsiRNAs mapped to the positive and negative strands of RGDV genome, respectively. Red and pink lines denote 22-nt vsiRNAs mapped to the positive and negative strands of RGDV genome, respectively. All reads in this analysis were redundant.

Figure S2 Characterizations of piRNAs 24-32 nt long in VCMs inoculated with RGDV at 72 hpi. (A) Size distribution of piRNAs. (B) Percentage of nucleotide at position 1 for antisense strands of 24-32 nt piRNAs. (C) Percentage of nucleotide at position 10 for sense strands of 24-32 nt piRNAs. All reads in this analysis are redundant.

Figure S3 Percentage of nucleotide at position 10 for sense strands (A) and at position 1 for antisense strands (B) of 18-32 nt vsiRNAs in VCMs inoculated with RGDV at 72 hpi. All reads in this analysis are redundant.

Figure S4 Mean viral genome copies in dead or live viruliferous leafhoppers treated with dsGFP or dsDCR2 from 9 day to 18 days padp. Second-instar nymphs of *R. dorsali* were microinjected with dsGFP (A) or dsDCR2 (B), then allowed to feed on RGDV-infected rice plants for 1 day. Mean viral genome copies were calculated as the log of the copy number/μg insect RNA from a pool of 10 live insects or total dead insects daily from 9 to 18 days padp by RT-qPCR. The mean RT-qPCR assay results ± standard deviation (SD) of three biological replicates are shown. * $P < 0.05$. Statistical analysis was conducted based on Tukey's honest significant difference (HSD) test using SAS version IV (SAS Institute, Cary, NC, USA).

Figure S5 The treatment of dsDCR2 significantly increased viral accumulation in the leafhoppers, as detected by RT-qPCR assay. Second-instar nymphs of *R. dorsali* (n = 100) were microinjected with dsDCR2 or dsGFP, then allowed to feed on RGDV-infected rice plants for 1 day. Viral titers were calculated as the log of the copy number/μg insect RNA from 10 live insects and 10 dead insects daily from 9 to 13 days padp by RT-qPCR. Each data point represents an individual insect. Horizontal lines represent the mean viral genome

copies for each data set. Black dots indicate live insects, red dots dead insects. * $P < 0.05$. Statistical analysis was conducted based on Tukey's honest significant difference (HSD) test using SAS version IV (SAS Institute, Cary, NC, USA).

Figure S6 The treatment of dsDCR2 significantly increased viral accumulation in the leafhoppers, as detected by immunofluorescence microscopy. Midguts and salivary glands of viruliferous leafhoppers treated with dsGFP (I) or dsDCR2 (II) were dissected, immunolabelled with viral particle-specific IgG conjugated to rhodamine (red) and the actin dye phalloidin-FITC (green) at 5 or 10 days padp. Fc, filter chamber; mg, midgut; sg, salivary glands. Bars, 70 µm.

Figure S7 Tansmission rates of viruliferous leafhoppers (n = 100) treated with dsDCR2 or dsGFP at 12 days padp. The mean ± SD from three biological replicates was shown. * $P < 0.05$. Statistical analysis was conducted based on Tukey's honest significant difference (HSD) test using SAS version IV (SAS Institute, Cary, NC, USA).

Supplementary Table Legends

Table S1. Viral genome copies in individual viruliferous leafhoppers treated with dsGFP or dsDCR2 from 9 to 18 days padp.

Table S2. Primers used in this study.

Figure S1

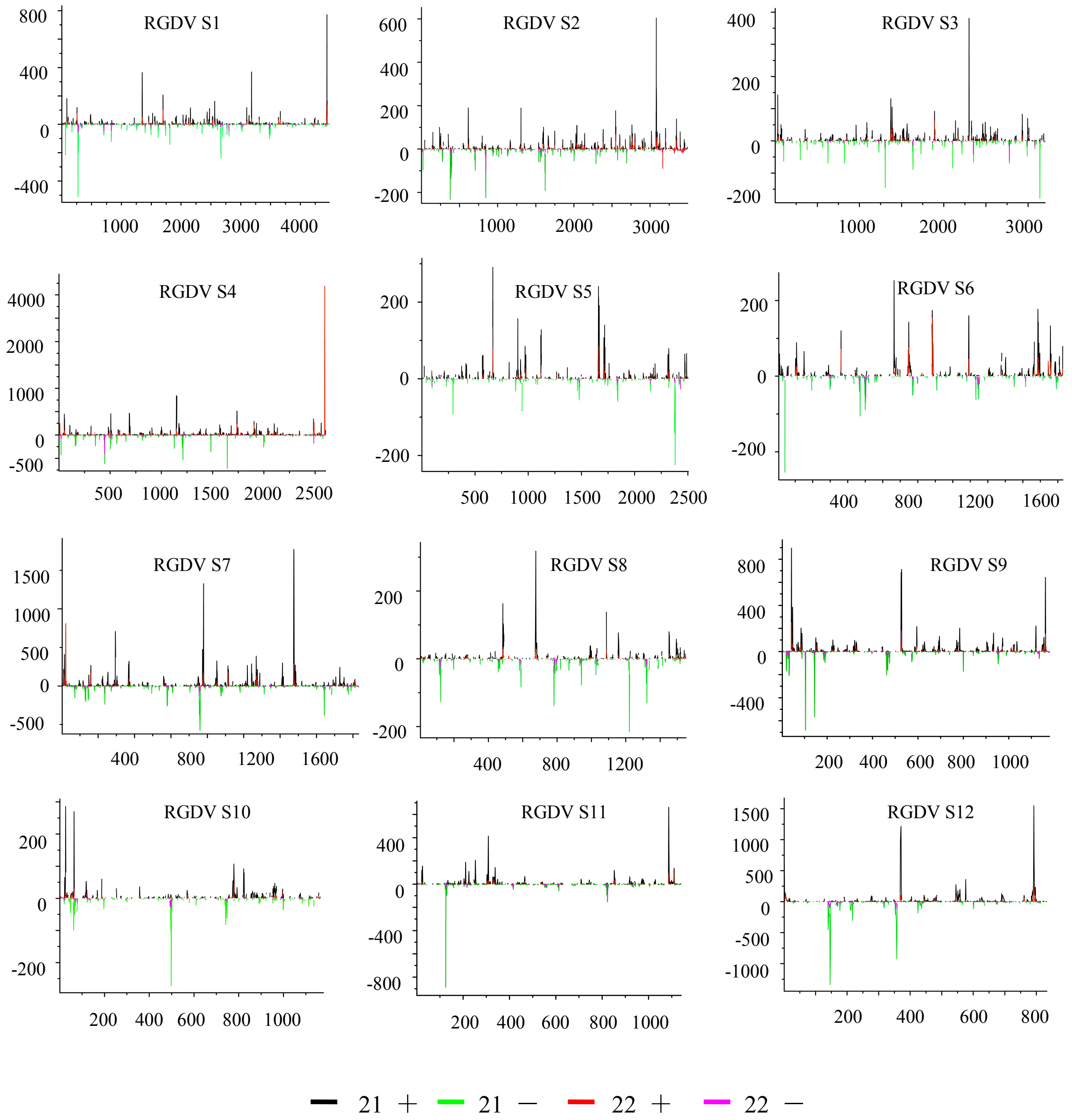
RGDV S1
RGDV S2
RGDV S3
RGDV S4
RGDV S5
RGDV S6
RGDV S7
RGDV S8
RGDV S9
RGDV S10
RGDV S11
RGDV S12
21 +
21 —
22 +
22 —

Figure S2

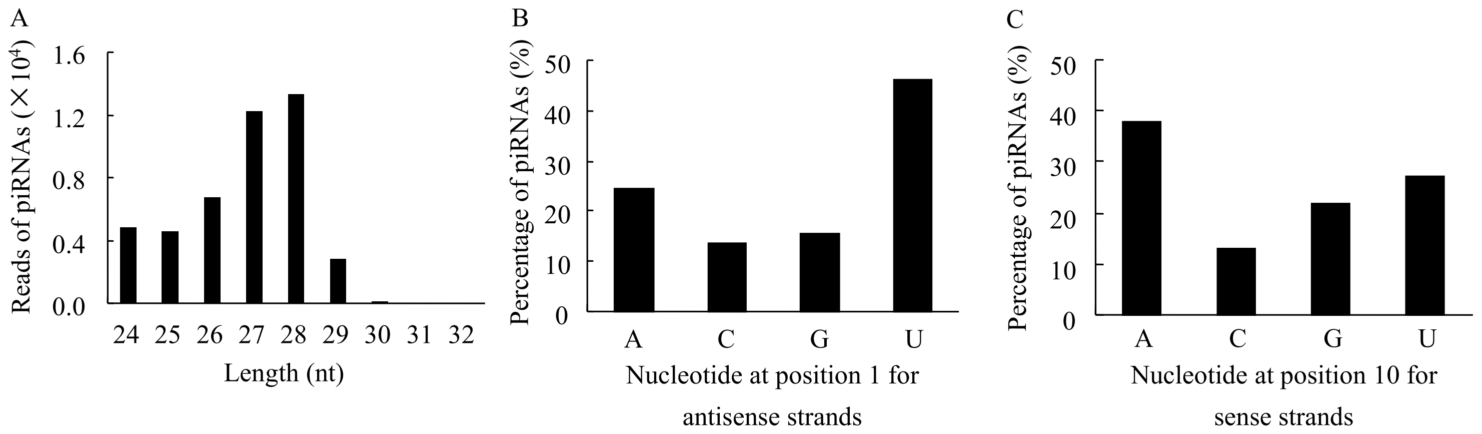

Figure S3

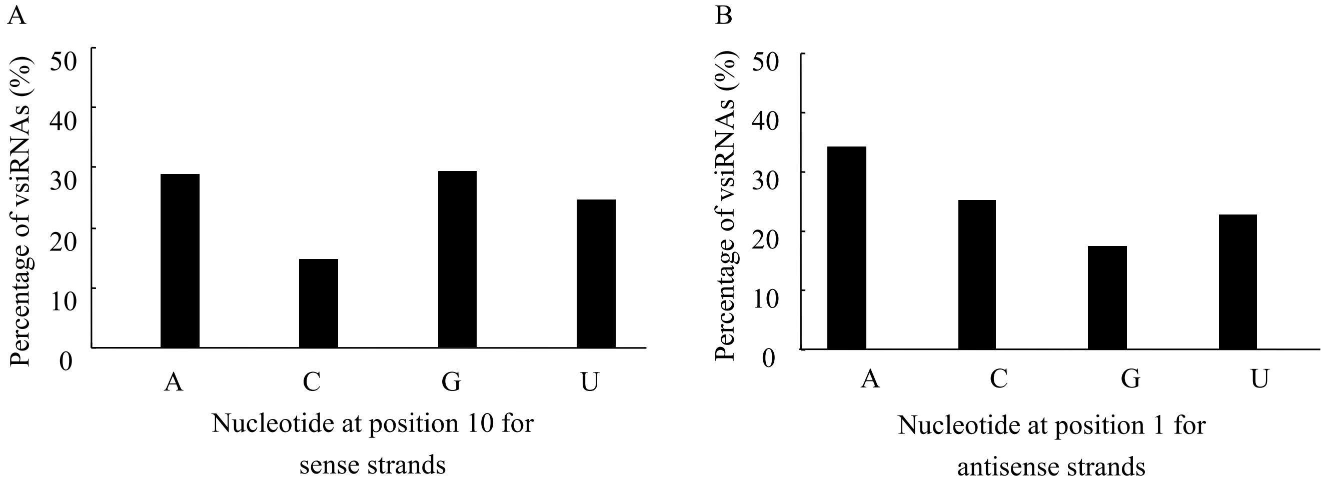

Figure S4

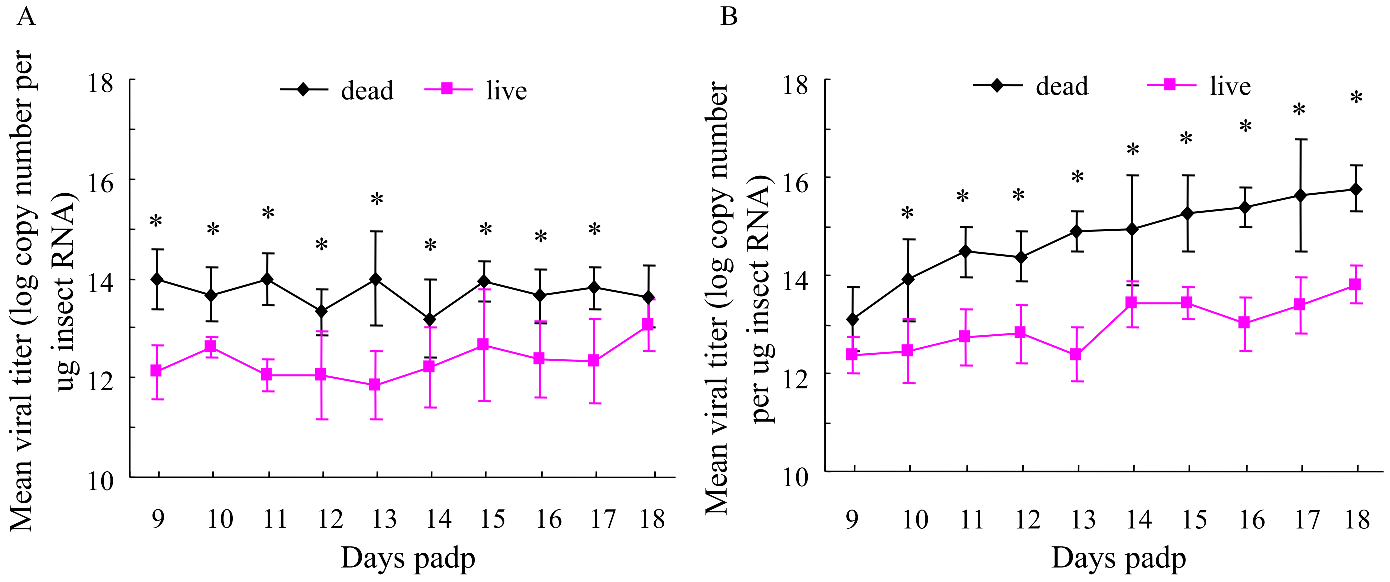

Figure S5

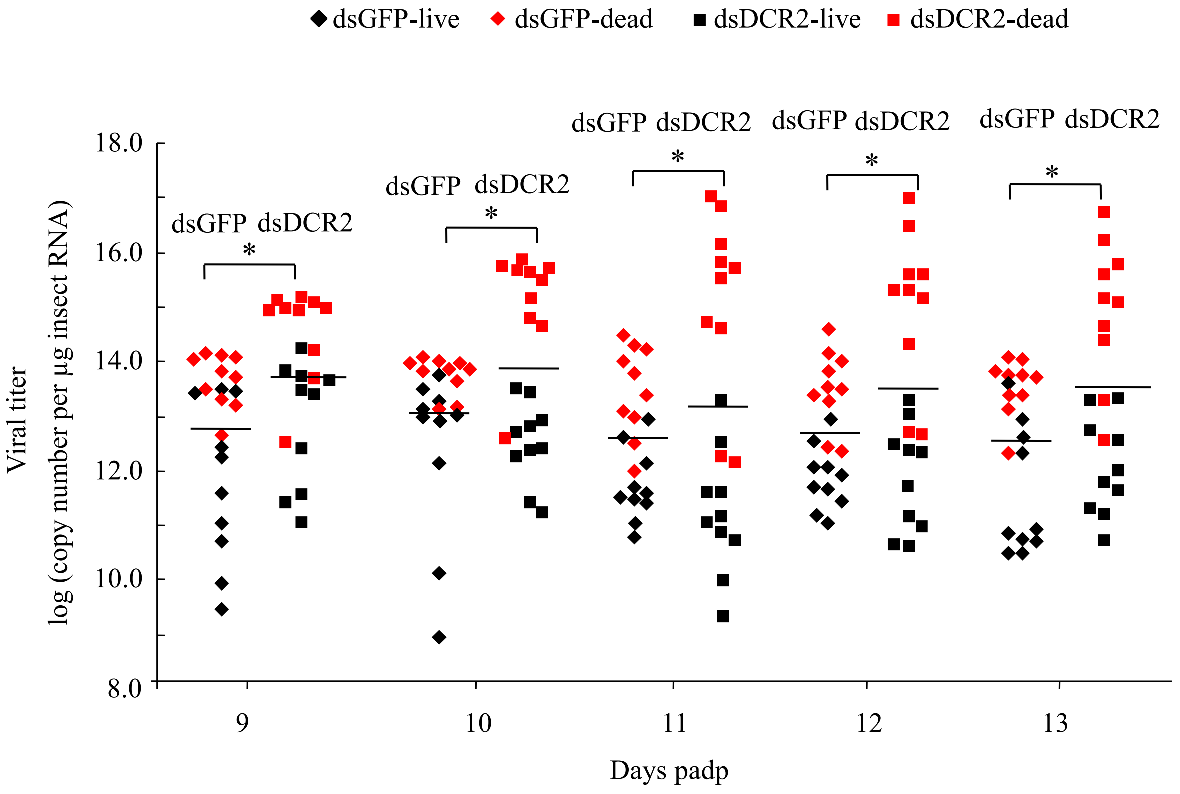

Figure S6

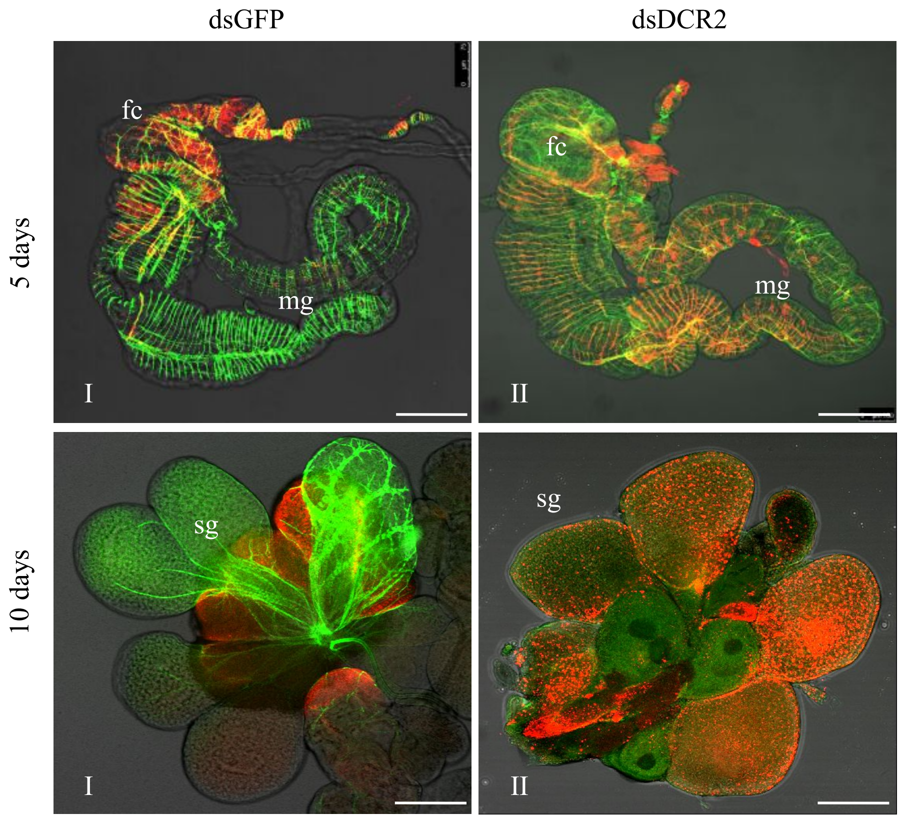

Figure S7

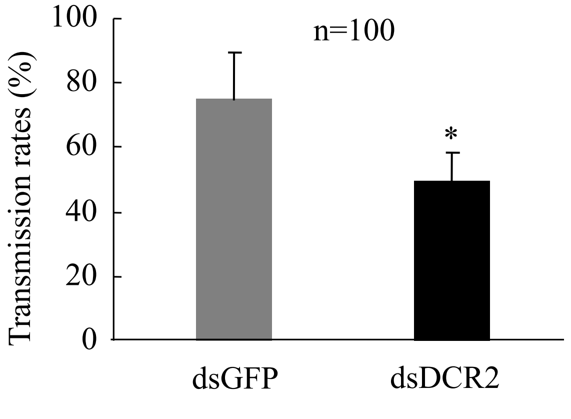

Table S1. Viral genome copies in individual viruliferous leafhoppers treated with dsGFP or dsDCR2 from 9 to 18 days padp.

Insect no.	Viral genome copies in individual viruliferous leafhoppers									
	9 days padp		10 days padp		11 days padp		12 days padp		13 days padp	
	dsGFP	dsDCR2	dsGFP	dsDCR2	dsGFP	dsDCR2	dsGFP	dsDCR2	dsGFP	dsDCR2
1	1.68×10^{14}	1.72×10^{15}	1.71×10^{14}	1.56×10^{16}	4.97×10^{14}	3.70×10^{15}	1.02×10^{14}	2.96×10^{15}	5.60×10^{14}	1.63×10^{16}
2	1.60×10^{14}	1.34×10^{15}	1.20×10^{14}	1.40×10^{16}	1.76×10^{14}	3.41×10^{15}	3.48×10^{13}	1.00×10^{15}	1.38×10^{14}	5.44×10^{15}
3	1.41×10^{14}	1.24×10^{15}	1.01×10^{14}	1.29×10^{16}	1.69×10^{14}	2.85×10^{15}	2.44×10^{13}	9.15×10^{14}	1.13×10^{14}	3.81×10^{15}
4	1.39×10^{14}	1.22×10^{15}	1.01×10^{14}	1.18×10^{16}	1.32×10^{14}	2.68×10^{15}	8.49×10^{12}	4.03×10^{14}	8.88×10^{13}	1.45×10^{15}
5	1.34×10^{14}	1.20×10^{15}	9.85×10^{13}	1.17×10^{16}	9.35×10^{13}	2.45×10^{15}	3.61×10^{12}	3.97×10^{14}	5.12×10^{13}	1.35×10^{15}
6	1.21×10^{14}	1.18×10^{15}	9.46×10^{13}	1.17×10^{16}	5.16×10^{13}	2.38×10^{15}	2.65×10^{12}	1.97×10^{14}	3.95×10^{13}	1.16×10^{15}
7	1.06×10^{14}	1.11×10^{15}	9.15×10^{13}	1.13×10^{16}	8.95×10^{12}	2.05×10^{15}	2.22×10^{12}	1.89×10^{14}	2.47×10^{13}	5.84×10^{14}
8	6.50×10^{13}	9.56×10^{14}	7.49×10^{13}	1.12×10^{16}	4.13×10^{12}	1.59×10^{15}	1.21×10^{12}	1.58×10^{14}	4.26×10^{12}	2.31×10^{14}
9	4.99×10^{13}	9.42×10^{14}	7.49×10^{13}	1.09×10^{16}	1.40×10^{12}	1.40×10^{15}	1.16×10^{12}	1.37×10^{14}	2.13×10^{12}	1.44×10^{14}
10	3.22×10^{13}	9.15×10^{14}	6.55×10^{13}	9.42×10^{15}	9.56×10^{11}	9.96×10^{14}	8.43×10^{11}	3.81×10^{13}	2.09×10^{12}	4.26×10^{13}
11	3.00×10^{13}	9.01×10^{14}	6.55×10^{13}	8.37×10^{15}	5.05×10^{11}	6.60×10^{14}	4.94×10^{11}	3.64×10^{13}	8.56×10^{10}	2.09×10^{13}
12	2.95×10^{13}	8.81×10^{14}	5.69×10^{13}	8.18×10^{15}	3.84×10^{11}	2.47×10^{14}	4.76×10^{11}	2.06×10^{13}	7.21×10^{10}	1.95×10^{13}
13	2.56×10^{13}	8.75×10^{14}	4.38×10^{13}	7.60×10^{15}	3.31×10^{11}	1.39×10^{14}	2.73×10^{11}	1.88×10^{13}	5.77×10^{10}	1.92×10^{13}
14	1.98×10^{13}	8.62×10^{14}	3.21×10^{13}	7.49×10^{15}	2.96×10^{11}	1.07×10^{14}	1.48×10^{11}	1.84×10^{13}	5.09×10^{10}	5.24×10^{12}
15	1.58×10^{13}	8.49×10^{14}	1.83×10^{13}	6.80×10^{15}	2.49×10^{11}	6.55×10^{13}	1.09×10^{11}	1.07×10^{13}	3.05×10^{10}	3.61×10^{12}
16	4.52×10^{12}	8.37×10^{14}	1.43×10^{13}	6.75×10^{15}	1.13×10^{11}	4.90×10^{13}	N/A	4.69×10^{12}	2.98×10^{10}	3.38×10^{12}
17	2.65×10^{12}	8.12×10^{14}	1.30×10^{13}	5.56×10^{15}	5.86×10^{10}	4.22×10^{13}	N/A	4.35×10^{12}	N/A	9.56×10^{11}
18	1.73×10^{12}	8.06×10^{14}	1.01×10^{13}	5.54×10^{15}	N/A	4.01×10^{13}	N/A	3.07×10^{12}	N/A	5.73×10^{11}
19	4.00×10^{11}	5.95×10^{14}	9.39×10^{12}	5.09×10^{15}	N/A	1.92×10^{13}	N/A	2.20×10^{12}	N/A	4.07×10^{11}
20	1.12×10^{11}	1.71×10^{14}	8.09×10^{12}	4.79×10^{15}	N/A	3.08×10^{12}	N/A	2.06×10^{12}	N/A	1.92×10^{11}
21	4.49×10^{10}	1.53×10^{14}	1.39×10^{12}	4.48×10^{15}	N/A	1.83×10^{12}	N/A	4.90×10^{11}	N/A	1.52×10^{11}
22	8.95×10^{9}	6.75×10^{13}	2.75×10^{11}	4.26×10^{15}	N/A	1.40×10^{12}	N/A	1.36×10^{11}	N/A	5.12×10^{10}
23	2.94×10^{9}	5.36×10^{13}	8.95×10^{10}	4.19×10^{15}	N/A	3.95×10^{11}	N/A	9.42×10^{10}	N/A	N/A
24	N/A	4.65×10^{13}	8.06×10^{10}	3.02×10^{15}	N/A	3.95×10^{11}	N/A	4.26×10^{10}	N/A	N/A
25	N/A	4.29×10^{13}	N/A	2.91×10^{15}	N/A	1.44×10^{11}	N/A	3.92×10^{10}	N/A	N/A
26	N/A	2.85×10^{13}	N/A	1.42×10^{15}	N/A	1.12×10^{11}	N/A	N/A	N/A	N/A
27	N/A	2.42×10^{13}	N/A	8.37×10^{14}	N/A	7.27×10^{10}	N/A	N/A	N/A	N/A
28	N/A	3.12×10^{12}	N/A	5.99×10^{14}	N/A	5.01×10^{10}	N/A	N/A	N/A	N/A
29	N/A	2.55×10^{12}	N/A	4.26×10^{13}	N/A	9.49×10^{9}	N/A	N/A	N/A	N/A
30	N/A	3.53×10^{11}	N/A	2.57×10^{13}	N/A	2.06×10^{9}	N/A	N/A	N/A	N/A
31	N/A	2.63×10^{11}	N/A	3.72×10^{12}	N/A	N/A	N/A	N/A	N/A	N/A
32	N/A	1.09×10^{11}	N/A	2.57×10^{12}	N/A	N/A	N/A	N/A	N/A	N/A
33	N/A	N/A	N/A	2.25×10^{12}	N/A	N/A	N/A	N/A	N/A	N/A
34	N/A	N/A	N/A	1.77×10^{12}	N/A	N/A	N/A	N/A	N/A	N/A
35	N/A	N/A	N/A	6.12×10^{11}	N/A	N/A	N/A	N/A	N/A	N/A
36	N/A	N/A	N/A	4.07×10^{11}	N/A	N/A	N/A	N/A	N/A	N/A
37	N/A	N/A	N/A	2.47×10^{11}	N/A	N/A	N/A	N/A	N/A	N/A

38	N/A	N/A	N/A	1.36×10^{11}	N/A	N/A	N/A	N/A	N/A	N/A
39	N/A	N/A	N/A	2.51×10^{10}	N/A	N/A	N/A	N/A	N/A	N/A
40	N/A	N/A	N/A	2.96×10^{9}	N/A	N/A	N/A	N/A	N/A	N/A
Mean value	5.42×10^{13}	6.20×10^{14}	5.27×10^{13}	5.21×10^{15}	6.69×10^{13}	8.29×10^{14}	1.22×10^{13}	2.61×10^{14}	6.40×10^{13}	1.39×10^{15}

Insect no.	14 days padp		15 days padp		16 days padp		17 days padp		18 days padp	
	dsGFP	dsDCR2	dsGFP	dsDCR2	dsGFP	dsDCR2	dsGFP	dsDCR2	dsGFP	dsDCR2
1	1.11×10^{13}	2.71×10^{15}	8.56×10^{13}	3.51×10^{15}	5.56×10^{13}	5.32×10^{15}	1.50×10^{14}	1.87×10^{15}	1.95×10^{14}	1.81×10^{15}
2	1.02×10^{13}	1.25×10^{15}	2.07×10^{13}	2.28×10^{15}	3.41×10^{13}	4.94×10^{15}	5.48×10^{13}	1.21×10^{15}	9.49×10^{13}	1.21×10^{15}
3	9.49×10^{12}	5.99×10^{14}	2.01×10^{13}	1.47×10^{15}	1.49×10^{13}	1.98×10^{15}	4.29×10^{13}	1.07×10^{15}	9.42×10^{13}	8.06×10^{14}
4	8.49×10^{12}	1.80×10^{14}	1.89×10^{13}	1.33×10^{15}	1.10×10^{13}	1.75×10^{15}	2.65×10^{13}	8.88×10^{14}	8.31×10^{13}	6.17×10^{14}
5	2.31×10^{12}	1.35×10^{14}	1.77×10^{13}	1.02×10^{15}	6.85×10^{12}	1.23×10^{15}	5.24×10^{12}	7.11×10^{14}	7.05×10^{13}	3.09×10^{14}
6	1.56×10^{12}	9.08×10^{13}	1.57×10^{13}	3.86×10^{14}	4.79×10^{12}	8.68×10^{14}	3.26×10^{12}	2.91×10^{14}	3.61×10^{13}	1.98×10^{14}
7	3.95×10^{11}	7.05×10^{13}	1.47×10^{13}	1.91×10^{14}	3.70×10^{12}	5.52×10^{14}	2.98×10^{12}	1.40×10^{14}	3.12×10^{13}	8.37×10^{13}
8	2.46×10^{11}	6.08×10^{13}	4.29×10^{12}	7.00×10^{13}	3.51×10^{12}	5.12×10^{13}	2.98×10^{12}	1.39×10^{14}	2.77×10^{13}	6.17×10^{13}
9	1.78×10^{11}	4.13×10^{13}	2.65×10^{12}	5.40×10^{13}	2.03×10^{12}	3.07×10^{13}	1.67×10^{12}	3.95×10^{13}	2.04×10^{13}	7.76×10^{12}
10	1.72×10^{11}	3.19×10^{13}	1.01×10^{11}	3.85×10^{13}	1.41×10^{12}	2.20×10^{13}	6.70×10^{11}	2.40×10^{13}	1.60×10^{13}	7.21×10^{12}
11	4.19×10^{10}	1.00×10^{13}	5.81×10^{10}	3.70×10^{13}	3.72×10^{11}	9.93×10^{12}	3.84×10^{11}	2.03×10^{13}	5.77×10^{12}	5.01×10^{12}
12	1.75×10^{10}	4.17×10^{12}	1.87×10^{10}	6.92×10^{12}	3.02×10^{11}	7.94×10^{12}	8.95×10^{10}	1.60×10^{13}	2.96×10^{12}	3.48×10^{12}
13	N/A	3.47×10^{12}	N/A	3.75×10^{12}	1.07×10^{11}	7.83×10^{12}	2.47×10^{10}	8.88×10^{12}	3.78×10^{11}	3.48×10^{12}
14	N/A	2.81×10^{12}	N/A	3.56×10^{12}	N/A	3.41×10^{12}	N/A	7.60×10^{12}	2.07×10^{10}	2.40×10^{12}
15	N/A	2.81×10^{12}	N/A	2.73×10^{12}	N/A	3.41×10^{12}	N/A	7.37×10^{12}	N/A	5.75×10^{11}
16	N/A	2.09×10^{12}	N/A	1.12×10^{12}	N/A	4.01×10^{11}	N/A	1.69×10^{12}	N/A	3.14×10^{11}
17	N/A	1.23×10^{12}	N/A	1.12×10^{12}	N/A	1.67×10^{11}	N/A	1.69×10^{12}	N/A	1.74×10^{11}
18	N/A	6.46×10^{11}	N/A	4.37×10^{11}	N/A	7.27×10^{10}	N/A	1.01×10^{12}	N/A	8.71×10^{10}
19	N/A	2.83×10^{11}	N/A	2.40×10^{11}	N/A	N/A	N/A	8.62×10^{11}	N/A	1.95×10^{10}
20	N/A	2.69×10^{11}	N/A	1.01×10^{11}	N/A	N/A	N/A	8.62×10^{10}	N/A	N/A
21	N/A	4.13×10^{10}	N/A	7.16×10^{10}	N/A	N/A	N/A	2.03×10^{10}	N/A	N/A
22	N/A	N/A	N/A	1.94×10^{10}	N/A	N/A	N/A	N/A	N/A	N/A
Mean value	3.69×10^{12}	2.47×10^{14}	1.67×10^{13}	4.73×10^{14}	1.07×10^{13}	9.32×10^{14}	2.24×10^{13}	3.07×10^{14}	4.85×10^{13}	2.70×10^{14}

Note: Green or orange shading denotes the dead insects according to treatment; nonshaded values are for live insects. N/A, no available.

Table S2. Primers used in this study.

Genes	Primers	Sequences (5′-3′)
RGDV-P8	Q-P8-forward	GATTCAAGGGGCACAGAACG
	Q-P8-reverse	GTAATGGTTGCGACTGGGTC
	P8-forward	ATGGACGTAGCTAGATCGTCA
	P8-reverse	ACACACTAACTACTAGCAATAGA
GFP	T7-GFP-forward	T7-ATGTGCTGCAACATGAGCTCAC
	T7-GFP- reverse	T7-TTACGCAAAGTACATGACTTTCTTG
Actin	Q-actin-forward	GCCGTCTTTCTTGGGTATGG
	Q-actin- reverse	GCCGTCTTTCTTGGGTATGG
Argonaute1	T7-AGO1-forward	T7-ATGTTCAACTGCCCCCGGC
	T7-AGO1-reverse	T7-TGTTGAGCATCATCTTCCACTGG
	Q-AGO1-forward	ACAAGCCACATCGCATCATC
	Q-AGO1-reverse	TGCCTCTTCTGAACCACGAT
Argonaute2	T7-AGO2-forward	T7- ATCTTCTACAGAGACGGCGTC
	T7-AGO2-reverse	T7- CTAAGTGAAGAACATCGGGGCT
	Q-AGO2-forward	AATCAGGATGACAGTGGGCA
	Q-AGO2-reverse	CTCTTGTAGCGACGGAAAGC
Dicer1	T7-DCR1-forward	T7-CATCGCGATATTCCTCAACTACA
	T7-DCR1-reverse	T7-TGCGGTTCACGTATCGAGGAGTTA
	Q-DCR1-forward	ACCAAGACCAGCCTCAGTAC
	Q-DCR1-reverse	AGAAAGTTGAGTCGAGCGGA
Dicer2	T7-DCR2-forward	T7-ATGGAAGAAAAAGACTTCATAGCA
	T7-DCR2-reverse	T7- CCTTTGTGACTGCCACATTCTTG
	Q-DCR2-forward	CCACATTTACGAGCGATGCA
	Q-DCR2-reverse	ATGTCGTTGAGTGAGCTGGA

T7 sequence: ATTCTCTAGAAGCTTAATACGACTCACTATAGGG.

T7 denotes primers used for RNA silencing.

Q denotes primers used for RT-qPCR
